# A Global and Spatially Explicit Assessment of Climate Change Impacts on Crop Production and Consumptive Water Use

**DOI:** 10.1371/journal.pone.0057750

**Published:** 2013-02-27

**Authors:** Junguo Liu, Christian Folberth, Hong Yang, Johan Röckström, Karim Abbaspour, Alexander J. B. Zehnder

**Affiliations:** 1 School of Nature Conservation, Beijing Forestry University, Beijing, China; 2 Ecosystems Services & Management Program, International Institute for Applied Systems Analysis, Laxenburg, Austria; 3 SIAM, Swiss Federal Institute of Aquatic Science and Technology, Duebendorf, Switzerland; 4 Stockholm Environment Institute, Stockholm, Sweden; 5 School of Biological Sciences, Nanyang Technological University, Singapore, Singapore; 6 Alberta Water Research Institute, Edmonton, Alberta, Canada; Texas Tech University, United States of America

## Abstract

Food security and water scarcity have become two major concerns for future human's sustainable development, particularly in the context of climate change. Here we present a comprehensive assessment of climate change impacts on the production and water use of major cereal crops on a global scale with a spatial resolution of 30 arc-minutes for the 2030s (short term) and the 2090s (long term), respectively. Our findings show that impact uncertainties are higher on larger spatial scales (e.g., global and continental) but lower on smaller spatial scales (e.g., national and grid cell). Such patterns allow decision makers and investors to take adaptive measures without being puzzled by a highly uncertain future at the global level. Short-term gains in crop production from climate change are projected for many regions, particularly in African countries, but the gains will mostly vanish and turn to losses in the long run. Irrigation dependence in crop production is projected to increase in general. However, several water poor regions will rely less heavily on irrigation, conducive to alleviating regional water scarcity. The heterogeneity of spatial patterns and the non-linearity of temporal changes of the impacts call for site-specific adaptive measures with perspectives of reducing short- and long-term risks of future food and water security.

## Introduction

Climate change, in addition to population increase, economic growth and shifting diets, is one important driving force influencing earth's food and water ecosystems, and its impacts have become a topic of increasing research attention [1], [2], [3], [4], [5], [6], [7], [8], [9]. With increasing scientific and political interest in prioritizing investment needs for climate change mitigation and adaptation, there is a strong impetus to identify climate impact hotspots on a global scale but with a high spatial resolution [4]. Understanding spatial patterns of climate change impacts on crop production and water use is necessary not only for identifying climate change hotspots but also for helping formulating adaptive and mitigating measures at all geographical levels [4]. Such spatial assessments have become possible with recent advances in information technology and modeling techniques, in particular, with the development of GIS supported biophysical and ecological models (e.g. GEPIC[10], LPJmL[11] and GCWM[12]).

There are large numbers of studies devoted to assessing impacts of climate change on future world agricultural production [3], [4], [13], [14], [15] and agricultural water use [7], [18], [19]. However, most global level analyses often have not made full use of the spatially explicit databases available to address uncertainties of the assessments stemmed from using different Global Climate Models (GCMs) as well as the emission scenarios. Meanwhile, they often provide aggregated results on the global, national or regional scales (e.g. [9], [15]) and rarely pay attention to the spatial variations within a country or region. Spatially explicit assessments still remain lacking for simultaneous analysis of changes in crop production and agricultural water use in the context of climate change.

In this study, we analyze the impacts of climate change on the production and water use of major cereal crops on a global scale with a spatial resolution of 30 arc-minutes (about 50×50 km^2^ near the equator) for the 2030s (short term) and the 2090s (long term), respectively. A GIS-based EPIC (GEPIC) biophysical crop model is applied for the investigation. The simulation is performed at the grid level. The results then are aggregated to national, continental and global levels to address broader implications. Three crops, i.e. wheat (Triticum aestivum L.), maize (Zea mays L.) and rice (Oryza sativa L.), are selected as representatives due to their importance for humans. They provide more than 60% of human dietary calorie intakes either as cereals for direct human consumption or as feed grains to produce livestock products [28]. These crops will continue to account for the bulk of the future human food supply because of their higher productivity, faster growth, easier way for storage and transportation, and less fuel and labor requirements for processing and cooking compared to other food crops [29].

## Materials and Methods

### 2.1 Crop production and consumptive water use (CWU)

The simulation of crop yield and evapotranspiration (ET) is performed with a GEPIC model [10]. EPIC is a biophysical crop growth model developed in the mid 1980s [16] and has been widely applied in the literature [17], [18], [19], [20], [21], [22]. The development of GEPIC extends the model's capacity for spatially explicit investigation. The GEPIC model has been well calibrated and validated on different geographical scales for the simulation of crop yield and production [10], [23] [24], [25] and for the simulation of ET [26], [27]. The simulation results from the GEPIC model are satisfactory for crop yield and ET [10], [23] and for irrigation depth on a global scale [26]–[27]. In addition, the simulated crop water productivity (the ratio of yield to ET) from the GEPIC model [10] shows a good correlation with measured values from a global literature review by Zwart et al. [28]. Zward et al. [29] also confirmed that their results of crop water productivity compare very well with the simulated results from GEPIC for most countries. Also EPIC itself has been validated and applied in several studies on climate change impacts on large geographical scales (e.g. van der Velde et al. [30]; Gaiser et al. [31]). The model has demonstrated a good performance in its application in different regions of the world [17], [18], [19], [20], [21], [22].

GEPIC consists of a crop growth module to calculate crop yield and a hydrology module to estimate crop ET [10], [23]. Crop yield is estimated by multiplying the aboveground biomass at maturity with a water stress adjusted harvested index [16]. Biomass is calculated on a daily basis by considering solar radiation, leaf area index, a crop parameter for converting energy to biomass, and several environmental stresses caused by water, nitrogen and phosphorus deficiencies, extreme temperatures, and poor soil aeration[16]. Actual crop ET is estimated based on the potential crop transpiration and soil water content and snow cover[16]. Reference crop ET is calculated with the Hargreaves method[32], which is a temperature based method and is widely used when climatological data is limited.

Crop production is calculated by multiplying crop yield by harvested area. CWU refers to the total evaporative use during crop growth period, and it is calculated by multiplying actual ET by harvested area [23].

An aggregated production index (API) and aggregated CWU index (AWI) are calculated as the total crop production and total CWU, respectively, of all representative crops under both rainfed and irrigated systems. 
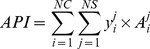
(1)

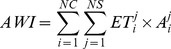
(2)


where *y* is crop yield in kg ha^−1^, *A* is harvested area in ha, *i* is crop code, and *j* is production system code (e.g. rainfed, irrigated), *NC* is the number of crops, and *NS* is the number of production systems. For wheat and maize, both rainfed and irrigated systems are considered. For rice, only irrigated systems are used due to their dominance in the production.

### 2.2 Irrigation water proportion

The CWU in the irrigated system consists of water from rainfall and irrigation. The irrigation water proportion in CWU is calculated as the ratio of the irrigation (consumptive) water use to the total CWU of all the representative crops under rainfed and irrigated systems. In order to quantify the irrigation water use in irrigated agriculture, a two-soil-water-balance approach is adopted as described in Liu et al. [26]. For this approach, in the first soil water balance, it is assumed that soil does not receive any irrigation water; while in the second soil water balance, it is assumed that soil received sufficient irrigation. Irrigation water proportion of a crop is calculated as the ratio of the difference of ET calculated in the two soil water balances to the ET calculated in the second soil water balance. The irrigation water use of the crop is calculated by multiplying the CWU of the crop by the irrigation water proportion. An aggregated irrigation water index (AIWI) is calculated by dividing the total irrigation water use of the representative crops by the AWI value.
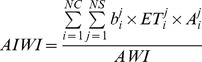
(3)


where *b* is the irrigation water proportion of crop *i* under production system *j*.

### 2.3 Impacts of climate change

For both the crop growth and hydrology modules embedded in the GEPIC model, climate variables (e.g. maximum temperature, minimum temperature and precipitation) are important inputs in addition to crop and soil parameters, and management practices. This enables the analysis of the impacts of climate change on crop production and consumptive green/blue water uses [33]. Here blue water use refers to ET that is fed by irrigation, while green water use is ET fed by unsaturated soil water received directly from precipitation. Three time periods are studied: the baseline period 1990s and two future periods 2030s and 2090s. The period of 2030s represents the near future, and this time period is most relevant to large agricultural investments, which typically take 15 to 30 years to realize full returns[34]. The time period of 2090s represents the far future, for which long-term effects of climate change are prominent.

Climate change scenarios are developed from the Intergovernmental Panel on Climate Change Special Report on Emission Scenarios (IPCC SRES) storylines[35] (emission scenarios hereafter). Results from four GCMs are used: HadCM3[36], CGCM2[37], CSIRO2 [38] and PCM [39]. These four GCMs are selected due to three reasons: First, they are standard GCMs, and commonly used in climate change impact studies; Second, high-resolution gridded data on monthly climate information have been generated based on outputs from these GCMs and on climatological observations [40], [41]; Third, for the SRES scenario A1FI, the CSIRO2 and HadCM3 models can be considered ‘hot’ models with temperature increases of up to 5.5 °C until the year 2100. PCM is rather ‘cold’ with a maximum increase of 3.5°C until 2100. CGCM2 is in the upper middle with up to 5 °C [40], [41], [42]. For each GCM, the two most socio-economically contrasting emission scenarios A1FI and B2 are selected; hence, there are eight scenarios for each crop-system combination. A1FI and B2 are selected in order to cover a wide range of possible developments of human society. A1FI is characterized by market globalization and reliance on fossil energy sources, while B2 assumes economic regionalization and use of mainly renewable energy. In terms of atmospheric CO_2_ concentration, A1FI has the highest CO_2_ concentration among all scenarios (e.g. 480 ppm in the 2030s and 928 ppm in the 2090s). In contrast, B2 has the lowest CO_2_ concentration before the middle of this century (e.g. 441 ppm in the 2030s); while afterward, it still remains a scenario with relatively very low CO_2_ concentration (e.g. B2 has the second lowest CO_2_ concentration next to B1 in all scenarios at the end of this century).

We first calculate API, AWI, and AIWI in the baseline period 1990s. We then simulate those variables under eight climate scenarios in each of the two future periods (i.e. 2030s and 2090s) by only allowing changes in temperature, precipitation and CO_2_ concentration while holding other influencing factors unchanged over time. The settings of climate parameters are described in detail in Liu [33]. Changes in harvested areas are not considered because the main purpose is to study the impacts of climate change (rather than the mixed effects of climate change, land use change, and changes of other socio-economic factors). In addition, the amount of arable land has not changed significantly in more than half a century, and it is unlikely to increase much in the future [1].

Impact ratio (IR) is used as an indicator for analyzing the impacts of climate change on the output variables (e.g. *p*) under different scenarios. IR is defined as the ratio of the output variable in each future time period (i.e. 2030s or 2090s) to that in the baseline period (i.e. 1990s). A value of IR higher than 1 indicates that climate change will lead to higher output variables in the future study period compared to those in the baseline period, while a value lower than 1 indicates that climate change will help reduce the magnitude of the variables [33]. Confidence level is classified into seven categories based on the IR values in the eight scenarios, as shown in Table 1.

**Table 1:** Definition of confidence level in this study

Confidence Level	Criteria
Increase with high confidence	IR*>1 in at least 7 scenarios
Increase with medium confidence	IR>1 in 6 scenarios
Increase with low confidence	IR>1 in 5 scenarios
Decrease with low confidence	IR<1 in 5 scenarios
Decrease with medium confidence	IR<1 in 6 scenarios
Decrease with high confidence	IR<1 in at least 7 scenarios
Increase/decrease mixed	Other conditions except for all above cretiria

* IR (impact factor; see section 2.3) is an indicator for analyzing the impacts of climate change on a variable (e.g. crop production), and it is defined as the ratio of the variable in a future time period (i.e. 2030s or 2090s) to that in the baseline period (i.e. 1990s).’

### 2.4 Uncertainties from GCMs and emission scenarios

Results of climate change impacts are subject to many uncertainties due to incomplete knowledge about the underlying geophysical processes of global change (GCM uncertainties) and due to uncertain future scenarios (emission scenario uncertainties) [43]. There are several complex methods to assess the magnitude of GCM and scenario uncertainties, including nonparametric methods such as kernel density estimation and orthonormal series methods [43]. In this paper, we use a straight-forward approach to get a rough estimate of these uncertainties with a relative difference (RD) index in order to allow for a quick comprehension of these uncertainties. Here the RD is used to compare two numbers (or simulation results), and it is calculated as
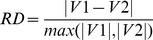
(4)


where V1 and V2 represent the two numbers that are compared, and *max* is the function for maximum value. Large *RD* numbers show high uncertainties.

For GCM scenario, we compare four different GCMs with the same scenario (e.g. CGCM2_A1FI vs. CSIRO2_A1FI), and this gives 12 RD values. For emission scenario uncertainty, we compare two emission scenarios (A1FI and B2) in each of the four GCMs, and this gives four RD values.

### 2.5 Data

The data on harvested area of wheat, maize and rice are obtained from the Center for Sustainability and the Global Environment (SAGE) of the University of Wisconsin at Madison, USA[44]. The SAGE data are spatially explicit and consistent with the statistical data from Food and Agriculture Organization of the United States (FAO). The harvested areas under irrigated systems of each crop are taken from the Institute of Physical Geography of the University of Frankfurt (Main), Germany [45]. The datasets from Portmann et al. [45] are currently the only source that provides high spatial resolution and crop-specific irrigated area at the global level. Both data sets are available with a spatial resolution of 30 arc-minutes. Water stress is measured with a withdrawals-to-availability ratio and the data are obtained from the Global Water System Project (GWSP) Digital Water Atlas [46].

Historical monthly climate data (maximum temperature, minimum temperature, precipitation, and number of wet days) for 1901-2002 were taken from the CRU TS2.1 database with a spatial resolution of 30 arc-minutes [41]. The TYN SC 2.0 dataset contains the climate projections of these four variables for 2003–2100 with a spatial resolution of 30 arc-minutes with the four GCMs (i.e. HadCM3, CGCM2, CSIRO2 and PCM) [41]. Monthly data are disaggregated to daily values with a weather converter MODAWEC[47]. The CO_2_ concentrations in different scenarios are obtained from the IPCC third assessment report[48]. Data sources for soil parameters and fertilizer application rates are identical to those in Liu et al.[26].

## Results

### 3.1 Impacts of climate change on crop production

**Figure 1:**
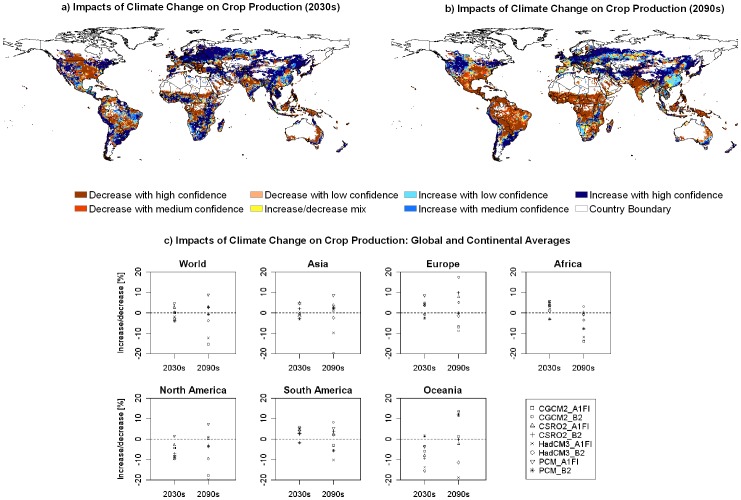
The impacts of climate change on crop production. (a) and (b) show the spatial distribution of confidence levels of increase or decrease for aggregated crop production index (API) in the 2030s and the 2090s, respectively, in comparison to the 1990s. (c) shows the relative change (%) of API caused by climate change on the global and continental scales.

Our results show significant spatial variations in the impacts of climate change on crop production across regions and among climate scenarios. Regarding the aggregated production index (API, the total amount of crop production of the three representative crops), climate change is likely to lead to higher API by the 2030s in a large part of Europe, northeast and western parts of the USA, northern China, southern Africa, the western and southeastern coastal areas of South America, while it will likely lead to lower API values in Southeast, East Central, Central, Midwest and North Central of the USA, the southern part of the cropland belt in Canada, the southern part of Europe, northern India, Southeast Asia, a large part of Australia, the south edge of the Sub-Saharan Africa, the central part of Africa, and a large part of the Amazon and Parana River Basins in South America (Fig. 1a). In the 2090s, the pattern of regional changes in crop production will become more evident. There is a general trend that high-latitude regions will have larger API values (except for Southeast and Midwest of the USA), whereas low-latitude regions have smaller API values (Fig. 1b). Higher crop production is likely to occur in the northern part of North America, western and southeastern coastal areas of South America, a large part of Europe, and the northern part of China, the southern part of Australia and New Zealand, while lower crop production is likely to occur in the southern part of North America, almost the entire Amazon and Parana River Basins, the dominant part of Africa, and most of India.

At the grid cell level, the projected impacts for the 2090s have a slightly lower agreement among the different scenarios than those for the 2030s. The grid cells with a high level of confidence, either for increase or decrease, account for 66.7% and 62.9% of the total grid cells simulated for the periods 2030s and 2090s, respectively.

At the global level, clear cut conclusions cannot be made on whether global crop production will increase or decrease in the future. Compared to the 1990s, the total crop production will change by −4.0% – +4.5% in the 2030s and −20.0% – +17.4% in the 2090s depending on different climate scenarios (Fig. 1c). A continental breakdown reveals considerable spatial heterogeneity across regions. In the 2030s, crop production in Africa and South America is likely to increase with a high level of confidence compared to that in the 1990s. In contrast, crop production in North America and Oceania is likely to decrease with a high level of confidence. Europe has more chances of higher crop production, while Asia does not have a clear trend in changes in crop production. In the 2090s, Africa and North America are likely to have lower crop production due to climate change, while in all other continent, trends in crop production are not clear, indicating high uncertainties of the impacts in these regions.

The country level projections are derived from aggregation of the grid cell simulation results (Table S1). During the period 2030s the crop production in 107 countries (out of the 166 countries studied) will benefit from climate change with different levels of confidence. Of which, 80 countries indicated an increase with a high level of confidence. Particularly, a large number of countries in African display an increase with a high level of confidence in the 2030s. However, during the period 2090s, the number of countries benefiting from climate change with a high level of confidence is reduced to 39. On the contrary, the number of countries with a high level of confidence for decrease rises from 33 in the 2030s to 55 in the 2090s. Many African countries turn to losses in crop production in the 2090s. Worldwide, a general trend is that the countries with high levels of confidence for increase in crop production in the 2030s remain an increase but with reduced levels of confidence. Many countries with low levels of confidence for increase in the 2030s tend to shift to decrease with low to high levels of confidence. The countries with the decreasing trend during the 2030s mostly remain the same trend during the 2090s. The results suggest a non-linearity of the impact of climate change over time (i.e. crop production is not directly proportional to climate parameters over time) for most of the countries. In general, the confidence level of the impact on crop production is lower during the period 2090s than the period 2030s. Crop production will increase or decrease with a high confidence level in 68.1% and 56.6% of the countries in the 2030s and the 2090s, respectively (Table S1).

### 3.2 Impacts of climate change on water use

Concerning the CWU for crop production, our results show that climate change will alter the magnitude of this variable and also the aggregated consumptive water use index (AWI, the total amount of consumptive water use for the representative crops) in cropland.

**Figure 2:**
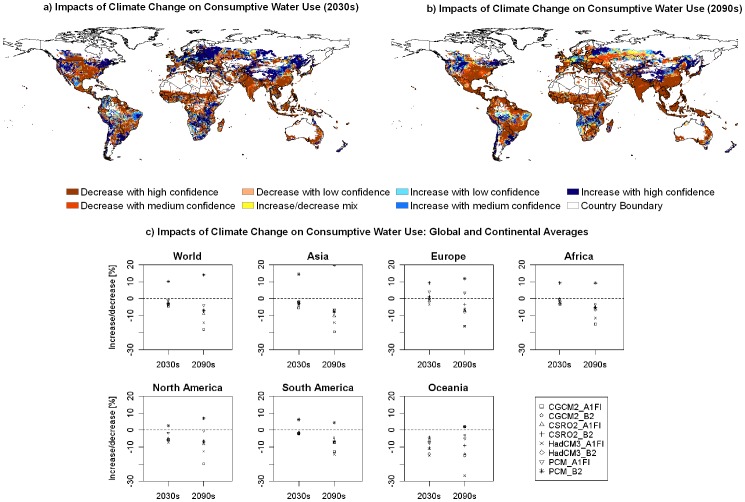
The impacts of climate change on consumptive water use (CWU). (a) and (b) show the spatial distribution of confidence levels of increase or decrease for aggregated CWU index (AWI) in the 2030s and the 2090s, respectively, in comparison to the 1990s. (c) shows the relative change (%) of AWI caused by climate change on the global and continental scales.

At the grid cell level, climate change will lead to lower AWI values in the 2030s with high and medium confidence levels in a large part of North America, West Africa and East Africa, India, North China Plain, southern parts of China, and Australia. In contrast, AWI will increase in northeast and the Great Basin in the USA, the coastal areas in the west and southeast of South America, a large part of Europe, the southern part of Africa, and the northern part of China (Fig. 2a). In the 2090s, the decreasing trend of AWI will become dominant with only a few regions remaining increasing trends (Fig. 2b). At the global level, climate change will reduce AWI. Compared to the 1990s, AWI will decrease in seven out of eight scenarios (except for the PCM_B2 scenario) for both the 2030s and 2090s (Fig. 2c). Climate change will reduce AWI values more significantly in the far future than in the near future. In the seven scenarios, AWI will decrease by 0.96–4.41% in the 2030s and 3.92–18.08% in the 2090s.

At the continental level, in the 2030s lower AWI occurs with a high level of confidence in all continents except for Europe which has no clear trend of increase or decrease (Fig. 2c). In the 2090s, all continents will have lower AWI. For Europe, lower AWI occurs in six out of eight scenarios; while in all the other continents, AWI decreases in seven out of eight scenarios. This indicates less amount of water (i.e. ET) will be consumed in cropland in the future. One possible reason for the lower AWI could be that higher CO_2_ concentration reduces crop stomatal closure thus decreases actual crop ET by reducing plant transpiration. This effect has been confirmed by several previous studies [49]–[50], and is one important reason for the decreasing AWI in our simulation.

**Figure 3:**
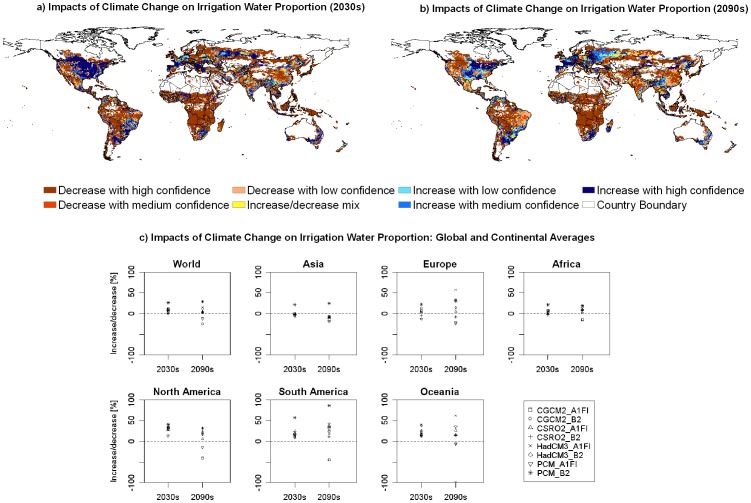
The impacts of climate change on irrigation water proportion. (a) and (b) show the spatial distribution of confidence levels of increase or decrease for aggregated irrigation water proportion index (AIWI) in the 2030s and the 2090s, respectively, in comparison to the 1990s. (c) shows the relative change (%) of AIWI caused by climate change on the global and continental scales.

Agricultural production is practiced in rainfed and irrigated systems. Under the irrigated system, crop uses both rainfall and irrigation water brought to the field. An aggregated irrigation water proportion index (AIWI) is calculated by dividing the total irrigation water use by the total consumptive water use of the representative crops. Our results indicate a general increase in AIWI in the future (Fig. 3a–b). On the global average, AIWI will increase with a high confidence level. Increase in AIWI occurs in seven out of eight scenarios with an increasing rate of 5.79–26.24%. The only exception is the A1FI scenario in PCM, which will lead to a slight decrease by 0.71%. In the 2090s, uncertainties are high on the global level. The change in AIWI will range between −25%–+29% on the world average.

Spatial heterogeneity exists among continents with a general decrease of AIWI in Asia, and general increase of AIWI in North America, South America and Oceania and Africa. In Europe, AIWI will increase in the southern parts while it will decrease in the northern parts in the 2030s (Fig. 3c). In the 2090s, Asia will have a lower AIWI with a high confidence level, Africa and South America will have a higher AIWI with high confidence level. For other continents, uncertainties are very high, and there are no clear trends of increase or decrease in AIWI.

**Figure 4:**
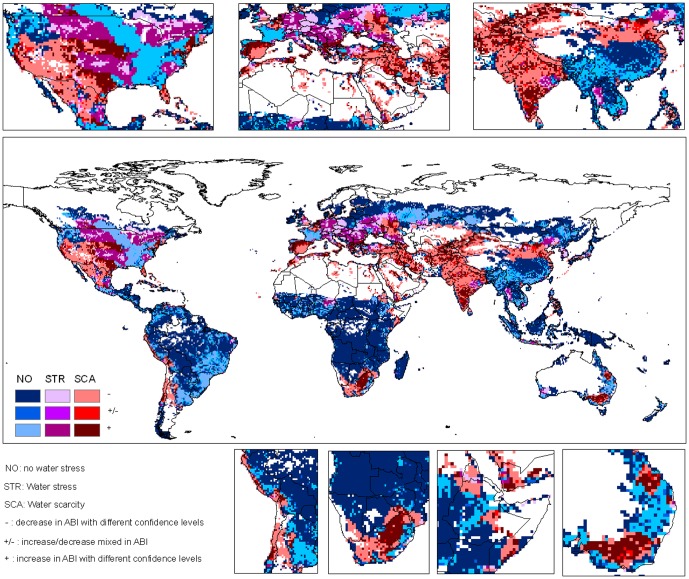
Change of irrigation water proportion in the 2030s in relation to water scarcity. Water scarcity is defined for the regions where total water withdrawal exceeds 40% of the freshwater resources, while water stress is defined for the regions where total water withdrawal is 20%–40% of the freshwater resources. *ABI* indicates irrigation water proportion.

**Figure 5:**
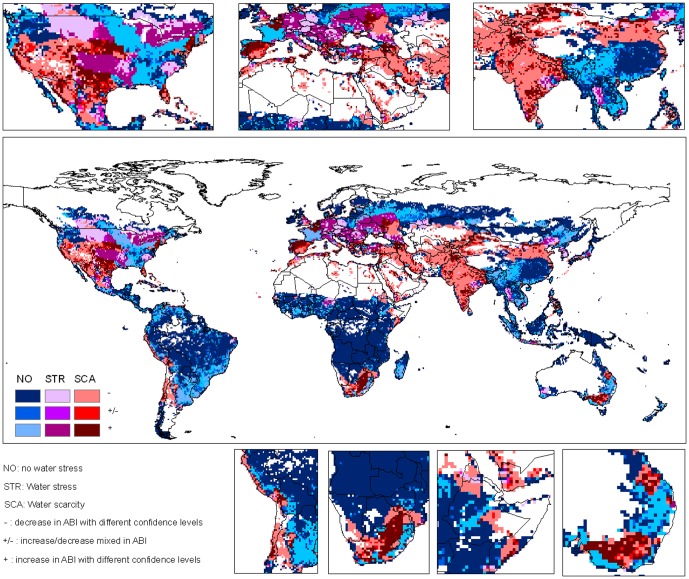
Change of irrigation water proportion in the 2090s in relation to water scarcity. Water scarcity is defined for the regions where total water withdrawal exceeds 40% of the freshwater resources, while water stress is defined for the regions where total water withdrawal is 20%–40% of the freshwater resources. *ABI* indicates irrigation water proportion.

The future hotspots of climate change impacts on freshwater ecosystems are most likely in the regions where water scarcity or stress problems already exist while irrigation water proportion will increase. These hotspots include the southern part of India, a large part of West Asia and the Mediterranean regions, a part of South Africa, and the Great Plains of the U.S. (Fig. 4 and 5). In these regions, crops will depend more on irrigation in the future as compared to the 1990s, though with different confidence levels. These regions are located mainly in developed countries or in rapidly developing areas e.g. in India, where mitigation and adaptation will more likely happen in these regions.

Despite the above hotspots, our results also show that many people in regions with current water scarcity problems will benefit from climate change. About 54.2% of the population who are currently suffering from water scarcity reside in the grid cells with reduced AIWI in the 2030s (Fig. 4). In contrast, 36.7% of the population in the water scarce regions will confront with higher dependency on irrigation. The findings that more people will benefit from climate change to alleviate water scarcity seem to conflict with the general increasing trend of AIWI at the global level. A close look indicates that the higher AIWI values occurs more often in regions where water is sufficient (e.g. in the Southeast of the U.S., Southeast of the South America etc) than the water scarce regions (Fig. 4 and Fig. 5). This spatial distribution results in more beneficiaries though the global AIWI has an upwards trend.

### 3.3 Latitudinal distribution of the impacts on crop yield and CWU

**Figure 6:**
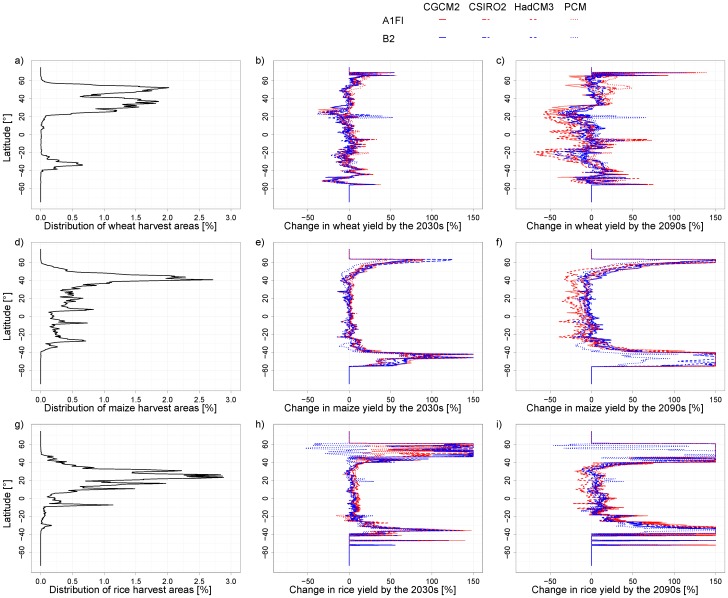
Latitudinal distribution of harvest area, and impacts of climate change on crop yield for wheat, maize and rice. A cut-off for changes in yield >150% is used to allow for a better interpretation of the impacts of climate change in current major growing regions where changes are mostly within a range of −50% – +50%. Cut-offs often occur at the latitudes where harvested areas are marginal currently.

The distribution of production of wheat, maize and rice varies in latitudinal gradients. Wheat is produced predominantly in temperate regions, maize in the sub-tropics and rice in the tropics (Fig. 6a, d, g). The impact of climate change in the short term (2030s) on wheat does not display a clear pattern with regard to latitudinal gradients (Fig. 6b). For maize, the large proportional increase is found in the high latitudinal areas, which are presently the marginal areas for maize production under the current climate (Fig. 6e). For rice, the relative increase is also mainly located in the high latitudinal marginal areas, while the magnitude of variations is substantial among different climate scenarios, particularly for the zone between 40^0^N–60^0^N (Fig. 6h). In the 2090s, variations among the different scenarios increase. Although the increase in crop yield remains for the higher latitude areas, the negative impact on the current major producing areas tends to become prominent (Fig 6c, f, i), particularly for wheat and maize.

**Figure 7:**
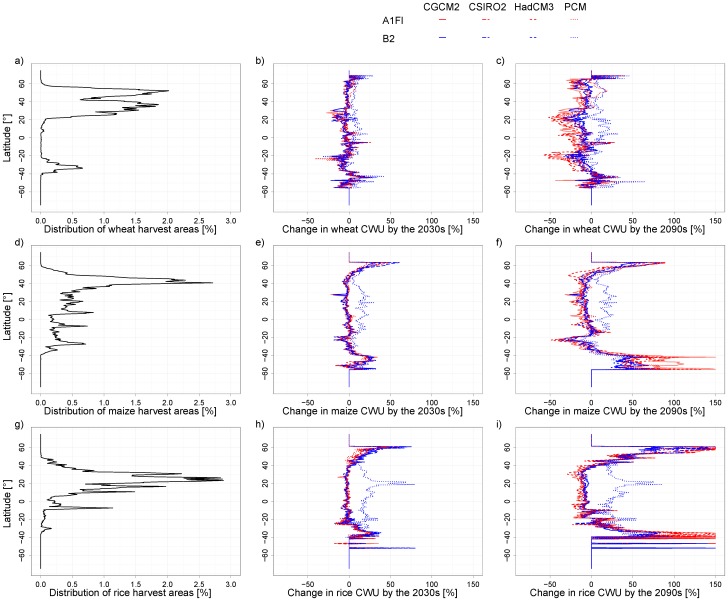
Latitudinal distribution of harvest area, and impacts of climate change on consumptive water use (CWU) for wheat, maize and rice. A cut-off for changes in CWU >150% is used to allow for a better interpretation of the impacts of climate change in current major growing regions where changes are mostly within a range of −50% – +50%. Cut-offs often occur at the latitudes where harvested areas are marginal currently.

Changes in CWU under climate change scenarios display similar patterns as to crop yields. In general, for the 2030s, the high latitudes outside the current major producing areas for the respective crops tend to have higher percentage increase in CWU (Fig. 7). For maize around 50°S, changes in the CWU in the 2030s are smaller than the changes in crop yield (Fig. 6 and 7). Cold weather there is a limiting factor for the growth of maize. In the future, temperature will become higher, leading to a more favorable climate and higher crop yield for maize. Nevertheless, the harvest areas for maize, as well as rice and wheat are currently marginal at this latitude. For the 2090s, the decrease in CWU is projected for many scenarios in the major producing areas for the respective crops. This is consistent with the lower crop yields resulted from climate change shown in Fig. 6. One exception is for the B2 scenario in PCM where the main producing areas of wheat, maize and rice are projected with significantly higher CWU, particularly for the 2090s.

### 3.4 Uncertainties in yield and CWU projections

**Figure 8:**
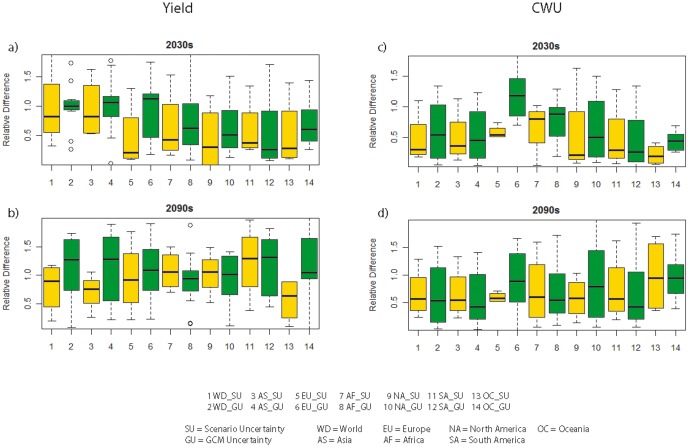
GCM and scenario uncertainties indicated by relative difference for crop production and CWU at global and continental levels in the 2030s and the 2090s.

In the short run (i.e. in the 2030s), the GCM uncertainties are generally higher than the emission scenario uncertainties (Fig. 8a). This applies to the results for five continents (Asia, Europe, Africa, North America and Oceania) as well as for the world as a whole. The only exception is South America, where GCM uncertainties are lower than that of the scenario uncertainties. In the long run (i.e. in the 2090s), the GCM uncertainties are higher than the scenario uncertainties for Asia, Europe, Oceania as well as the world, while contrasting situations occur for Africa and North America. The magnitudes of GCM and emission scenario uncertainties are similar for South America (Fig. 8b). For CWU (Fig. 8c,d), the global simulation shows a smaller uncertainty for the emission scenarios than for GCMs for both the short run and long run. The patterns, however, vary largely across different regions in terms of the relative magnitude of uncertainties from GCMs and emission scenarios.

## Discussion

### 4.1 Comparison with other studies

At the pixel level, we compare our results for crop production with two studies, which also demonstrate spatial patterns of the impacts of climate change on crop yield at a global level with high spatial resolutions. Deryng et al. [51] used a crop growth model PEGASUS and presented the impacts of climate change on crop yield of maize and spring wheat in the 2050s in comparison to 1961–1990 with a spatial resolution of 10 arc-minutes. They presented results for two conditions: fixed planting/harvest dates and planting/harvest dates allowed changing. The later condition is similar to our assumption which allows adapting planting/harvest dates with an automatic calendar algorithm [26]. So we only compare our results for the 2030s with those of the second condition. We find that our results for maize in general compare very well with those from Deryng et al. [51]. Both the studies show increasing maize yield in the high-latitude regions in the Northern Hemisphere. They also show an increasing trend in maize yield in western part of North America, western coastal areas of South America, Northern parts of Europe, northern parts of Asia, and eastern coastal areas of Australia. Meanwhile, they both show a decreasing trend in the southeast and Midwest of the US, the Amazon basin, Southern parts of Europe, a large part of India (except the northwest), and many Central African countries. The large differences exist mainly in a large part of Africa i.e. southern Africa, Eastern Africa, and western Africa, where increasing trends happen in many grid cells in our study, but decreasing trends generally occur in many pixels in Deryng et al. [51]. For wheat, large discrepancies exist between these two studies. We simulate for both spring wheat and winter wheat, while Deryng et al.[51] only simulated for spring wheat, and excluded regions where winter wheat is dominant. Since winter wheat is dominant compared to spring wheat on a global scale, it may be not easy to compare our results with Deryng et al. [51].

Tatsumi et al. [52] used an Improved Global Agro-Ecological Zones (iGAEZ) model to simulate the impacts of climate change on cereal yields in the 2090s compared to the 1990s with a spatial resolution of 30 arc-minute. Our results show general agreements with Tatsumi et al. for the impacts of climate change. For maize, agreements are found in most parts of North America, Africa, Australia, India, China, northern Asia and Russia, East Europe and Western Europe, while disagreements are mainly located in Central Europe and South America. For wheat, agreements are found in North America (except for Mid-west), South America, most parts of Africa, Australia, India, Northern Asia and Russia, while disagreements mainly occur in China, Europe and mid-west of the US. For rice, agreements are in North America, Western coastal regions of South America, Southern Africa, East Africa, West Europe, northern Asia and Russia, and China, while disagreements are mainly located in eastern coastal regions in South America, West Africa, East Europe, India and Australia. Besides the different approaches, another reason causing the disagreements may be the fact that Tatsumi et al.[52] only used one scenario (A1B). For example, for rice, our results indicate lower crop production in Southeast Asia with lower API values in most scenarios, while Tatsumi et al.[52] indicated higher crop production under A1B. According to the study by Babel et al.[53] in Thailand, an important rice producing country in Southeast Asia, the future climate change is likely to decrease the crop yield and production. The results from Babel et al.[53] agree well with our findings.

We also compare our results with other studies for China and India. For China, several crop models have predicted that cereal yields will increase under future climate change when the fertilizing effect of elevated CO2 is taken into account [54]–[56]. According to a comprehensive study by Xiong et al., [55], cereal production in 2050 will increase by 13%–22% relative to the average of 1961-1990 in China. Our results are consistent with these studies. For India, Aggarwal [57] reviewed and concluded that crop production will be reduced by 10–40% by 2100. Our simulations show a general decreasing trend of crop production in India with lower API values of −33.4% – −7.5% in the 2090s (Table S1).

For Africa, our results suggest that crop production will increase in the near future (e.g. the 2030s) but decrease in the far future (e.g. the 2090s). The results for the far future are consistent with the projections of the IPCC Synthesis Report (AR4), but those for the near future conflicts with the report. For Africa, a key conclusion in the IPCC AR4 was that ‘[b]y 2020, in some countries, yields from rain-fed agriculture could be reduced by up to 50% …would further adversely affect food security and exacerbate malnutrition’ [58]. This statement, however, is criticized for its nature of the underlying science (e.g., lack of sufficient scientific evidence from peer-reviewed literature) and procedural issues (e.g., whether the knowledge contained in the underlying scientific literature was properly represented on all levels of the report) [59]. Müller et al. [59] provide a comprehensive review for recent available literature based on multiple numbers of crops, and report a large uncertainty with no clear trend of lower crop yield in the 2030s. For Africa in the 2030s, three references were cited: Thornton et al. [60] for maize and beans; Liu et al.[33] for six major crops including wheat maize, and rice; and Lobell et al.[4] for 15 major crops also including wheat, maize and rice. Both Thornton et al. [60] and Liu et al.[33] indicated higher crop production in Africa, whereas Lobell et al.[4] show lower crop production. Another independent study, Adejuwon [61], also projected positive impacts of climate change on crop yields in Africa based on simulations for maize, rice, cassava, sorghum and millet. Hence, it still remains questionable for scientific robustness of the IPCC statement of negative effects of climate change on crop production in Africa in the near future.

### 4.2 Spatial Neutral Effect

The projection of climate change impacts on crop production and water use show lower uncertainties at smaller spatial scales (grid cell and country) than at higher spatial scales (continent and globe). The ‘spatial neutral effect (SNE)’ is an important reason for the high uncertainties on large geographical scales. SNE here means that trends that are displayed in small geographical scales are neutralized by aggregations and they become less obvious when observed on large geographical regions. For example, at the grid cell level, there is an increasing trend of crop production with a high confidence level for most grid cells at the coastal areas of Chile, Peru, Argentina, Uruguay and Brazil, but a contrastingly decreasing trend for many other regions in South America in the 2090s (Fig. 1b). However, the spatial neutral effect leads to high uncertainties when results are aggregated at the continental level (Fig. 1c). At the national scale, the confidence level of increase or decrease in crop production is relatively high. However, for some large countries like China, the general increasing trend of crop production hides the spatial variations among the country, e.g. the decreasing trend in the Guangdong province (southern part of China) in the 2030s. The generally higher confidence level at the smaller scale facilitates policy makers and investors to formulate adaptive and mitigation measures without being puzzled by a highly uncertain future on the global scale. The SNE phenomenon implies that climate change impacts should be assessed with high spatial resolutions to gain more in-depth insights.

### 4.3 Benefit in the Short Run, Prepare for the Long Run

Our study presents a generally less pessimistic perspective for climate change impacts in the short run than many other studies (Fig. 1, Fig. 7). For the short run, the climate change scenarios mostly projected small change in the yields in the current major producing areas of wheat, maize and rice. The major change occurs in the high latitude areas, which are outside of the current major producing zones for the respective crops. Hence, the climate change in the short run will benefit the high latitude areas in term of crop production. In the long run, the projections show large variations among climate scenarios. There is a high level of confidence for a decrease in the yields of wheat and maize in the currently major producing areas, although the increase remains for the high latitude areas. The projected increases in high latitude areas can be in part explained by the temperature increase there [30]. The projected large percentage increase in these areas suggests that the future climate change will lead to a geographical shift of major production areas of the three crops to currently marginal areas.

For Africa, the results show that the crop production is likely to benefit from climate change with a high level of confidence in the 2030s (Fig. 1). However, the continuous increase in temperature will lead to losses of crop production in the 2090s. The positive impacts in the short run can help alleviate food shortage problems. However, they may distract the attention paid to adapting and mitigating measures to combat the long-term negative impacts of climate change. A long-lasting effort is needed for the world to increase resilience to climate change and reduce the risks of future food and water security.

Impacts of climate change on Africa's crop production are often a scientific and policy concern. A more rigorous analysis is needed to assess the impacts for Africa, particularly by integrating simulations from a combination of a few models, literature reviews, and expert judgments including indigenous knowledge.

### 4.4 Uncertainties

We demonstrate the uncertainties from GCMs and emission scenarios concerning the impacts of climate change on crop production in the 2030s and the 2090s (Fig. 8). For the 2030s, the GCM uncertainties are generally higher than the emission scenario uncertainties. This indicates the importance of selecting multiple GCMs rather than a single GCM to analyze climate change impacts. For the 2090s, the GCM uncertainties and the emission scenario uncertainties appear heterogeneous across continents. Hence, in the long run, both the GCM and emission scenario uncertainties are important for analyzing impacts of climate change on crop production. The increasing emission scenario uncertainties stems from the difficulties in projecting the climate in the far future. To assess the climate change impacts, it is necessary to select multiple GCM as well as scenarios due to the inherent uncertainties among them.

### 4.6 Limitations of this study

Given the current absence of simultaneous simulation of impacts of climate change on crop production and consumptive water use with high spatial resolution, we consider the results from this paper encouraging and reasonable as an early approximation. Nonetheless, a number of limitations in our methodology and results still remain, and further research is needed in the future. First, we only provide simulation result with one crop model, GEPIC. Although the model has been validated at the global, continental and national scales in several previous studies[10], [26], [27], [33], [62], simulation results may be constrained by the fundamental assumptions and approaches used in this model. This shortcoming can be overcome by comparing results from several crop growth models, which use the same combination of climate, soil, land use, management and other input data. An intercomparision of different models is currently being conducted in the Agricultural Model Inter-comparison and Improvement Project (AgMIP) (http://www.agmip.org/). Second, the results of this study may be influenced by the lack of several spatially explicit data such as crop-specific fertilizer application rates, and crop-specific planting and harvesting data. Third, an unequivocal validation of our results for CWU is difficult because this article provides an early comprehensive assessment of the impacts of climate change on CWU. Forth, we have used a weather generator to disaggregate monthly data into daily data, and this disaggregation may give additional uncertainty. However, to the best of our knowledge, daily weather data are not yet available at the global level with a spatial resolution of 30 arc-minutes. There is a need for further improvements of spatially explicit daily weather data, but this is beyond our capacity as well as the scope of this study.

## Supporting Information

Table S1 **Ranges and confidence levels of the impacts of climate change on aggregated production index (API), aggregated consumptive water use index (AWI), and aggregated irrigation water proportion index (AIWI).**
(PDF)
