# Supplementary material for: A Global and Spatially Explicit Assessment of Climate Change Impacts on Crop Production and Consumptive Water Use
Source: PLoS One. 2013 Feb 27;8(2):e57750. doi: 10.1371/journal.pone.0057750 (PMC3583897; doi:10.1371/journal.pone.0057750)
Supplement: Table S1 — Ranges and confidence levels of the impacts of climate change on aggregated production index (API), aggregated consumptive water use index (AWI), and aggregated irrigation water proportion index (AIWI). (PDF) [file pone.0057750.s001.pdf]

Table S1. Ranges and confidence levels of the impacts of climate change on aggregated production index (API), aggregated consumptive water use index (AWI), and aggregated irrigation water proportion index (AIWI)

Note:

(1) A positive pencentage (e.g. +7.2%) shows that climate change will lead to an increase of the output variable (by 7.2%), while a negative percentage (e.g. -19.8%) shows that climate change will lead to a decrease of the output variable (by 19.8%);

(2) The subscripts of 30 and 90 indicates the periods of the 2030s and 2090s, respectively;

(3) ΔAPI_{30} indicates the relative change of API (in percentage) in the 2030s compared to the 1990s;

(4) The colors indicate different confidence levels of the impacts of climate change as follows:

Increase with high confidence

Increase with medium confidence

Increase with low confidence

Increase/decrease mixed

Decrease with low confidence

Decrease with medium confidence

Decrease with high confidence

Countries where no irrigation is applied (AIWI=0)

Country Name	Continent	ΔAPI_{30}	ΔAPI_{90}	ΔAWI_{30}	ΔAWI_{90}	$\Delta AIWI_{30}$	$\Delta AIWI_{90}$
Algeria	Africa	-19.8% - 7.2%	-16.0% - 23.2%	-14.5% - 0.5%	-26.8% - 1.4%	-7.2% - 84.6%	-18.1% - 81.4%
Angola	Africa	-6.0% - 5.8%	-5.1% - 11.2%	-1.2% - 9.8%	1.3% - 26.5%	-16.1% - 14.4%	-44.0% - 4.2%
Benin	Africa	-2.1% - 2.3%	-16.9% - -2.5%	-3.3% - 20.7%	-10.4% - 23.8%	-32.7% - 0.3%	-77.9% - 81.8%
Botswana	Africa	-2.8% - 11.4%	-29.5% - 6.2%	-10.5% - 4.7%	-32.9% - -8.8%	5.2% - 19.7%	-41.9% - 56.6%
Burkina Faso	Africa	-0.1% - 5.8%	-19.0% - -4.6%	-6.4% - 12.1%	-12.7% - 14.4%	-21.9% - 105.7%	-51.0% - 114.6%
Burundi	Africa	0.7% - 4.5%	-9.2% - 4.5%	-3.7% - 6.1%	-13.2% - 5.9%	-17.7% - 20.0%	-45.2% - 34.7%
Cameroon	Africa	1.6% - 5.8%	-16.1% - 0.0%	-4.1% - 20.5%	-12.5% - 22.3%	22.3% - 225.6%	-17.5% - 238.4%
Central Africa Republic	Africa	-6.0% - 1.1%	-30.1% - -3.2%	-1.5% - 18.5%	-8.9% - 16.6%	-15.9% - 338.9%	30.2% - 2396.0%
Chad	Africa	-6.4% - 0.7%	-21.5% - -0.8%	-6.3% - 5.6%	-14.4% - 8.2%	-5.0% - 32.9%	-36.6% - 23.6%
Comoros	Africa	-0.8% - 8.7%	-9.6% - 5.6%	-14.5% - 18.2%	-31.7% - 27.9%	0.0% - 0.0%	0.0% - 0.0%

Congo	Africa	-5.9% - 0.0%	-21.5% - -4.0%	-8.2% - 13.3%	-16.4% - 16.8%	0.0% - 0.0%	0.0% - 0.0%
Egypt	Africa	-6.9% - 4.4%	-46.6% - -1.3%	-4.8% - 18.1%	-18.6% - 18.6%	-0.6% - 1.8%	-3.9% - 18.9%
Equatorial Guinea	Africa	-5.2% - -2.3%	-14.9% - -5.2%	-8.4% - 24.3%	-22.0% - 35.3%	0.0% - 0.0%	0.0% - 0.0%
Eritrea	Africa	-9.9% - 1.3%	-15.0% - 15.9%	-5.5% - 1.9%	-21.3% - 13.7%	-26.4% - 22.3%	-86.1% - 2.2%
Ethiopia	Africa	-1.6% - 1.5%	-17.5% - 0.3%	-10.0% - 9.4%	-29.8% - 6.3%	-7.7% - 52.8%	-41.8% - 112.8%
Gabon	Africa	-6.5% - -0.6%	-13.9% - -4.3%	-14.0% - 20.3%	-19.1% - 26.9%	-41.7% - 62.4%	-29.2% - 171.0%
Gambia	Africa	-6.1% - -0.4%	-35.3% - -3.8%	-8.0% - 5.8%	-12.3% - 9.2%	-32.2% - 5.9%	-60.0% - 69.8%
Ghana	Africa	0.8% - 2.9%	-15.7% - -4.1%	-3.9% - 18.7%	-11.9% - 21.8%	-0.9% - 136.4%	-33.2% - 803.4%
Guinea	Africa	-0.8% - 1.9%	-18.0% - 1.0%	-7.1% - 20.9%	-15.0% - 26.8%	-33.8% - 179.7%	-46.4% - 193.9%
Guinea-Bissau	Africa	-0.7% - 4.1%	-25.6% - 3.5%	-8.2% - 25.6%	-16.2% - 30.8%	-33.8% - 65.2%	-6.7% - 197.4%
Ivory Coast	Africa	0.0% - 4.0%	-16.1% - -1.3%	-4.3% - 14.2%	-12.5% - 17.0%	-32.9% - 222.9%	-47.5% - 418.3%
Kenya	Africa	-1.0% - 6.3%	-11.3% - 3.9%	-0.1% - 8.7%	-7.4% - 7.2%	-20.2% - 17.6%	-44.8% - 16.0%
Lesotho	Africa	24.5% - 30.6%	20.1% - 47.5%	6.8% - 12.9%	-3.9% - 24.8%	-22.2% - 29.9%	-67.0% - 125.1%
Liberia	Africa	-0.5% - 3.7%	-19.9% - -5.7%	-4.1% - 18.4%	-14.3% - 24.7%	10.6% - 1299.6%	96.4% - 1127.8%
Libya	Africa	-26.1% - 6.7%	-22.9% - 13.2%	-6.5% - -0.6%	-19.3% - -1.0%	-14.1% - 42.8%	-12.2% - 45.1%
Madagascar	Africa	-0.1% - 10.4%	5.7% - 17.7%	-6.2% - 21.6%	-5.4% - 30.7%	-26.1% - 3.3%	-7.5% - 28.9%
Malawi	Africa	1.5% - 16.1%	-4.8% - 16.6%	3.5% - 19.4%	-0.7% - 19.4%	-35.8% - -10.9%	-77.9% - -18.1%
Mali	Africa	-3.7% - 3.4%	-27.1% - -0.3%	-7.4% - 10.9%	-15.0% - 17.0%	-13.8% - 19.2%	-51.9% - 16.8%
Mauritania	Africa	-11.0% - 4.4%	-44.8% - 2.2%	-9.9% - 2.5%	-19.0% - 7.9%	0.5% - 16.7%	-13.8% - 34.8%
Morocco	Africa	-18.2% - 8.3%	-19.0% - 11.1%	-5.3% - -0.5%	-16.1% - 2.6%	-8.7% - 65.2%	-23.5% - 52.3%
Mozambique	Africa	-5.2% - 8.9%	-15.0% - 8.0%	-5.7% - 17.7%	-11.0% - 8.8%	-19.3% - 15.8%	-63.8% - 12.0%
Namibia	Africa	-4.4% - 18.3%	-47.8% - 36.5%	-12.6% - 27.3%	-41.9% - 42.8%	-10.4% - 11.7%	-95.0% - 27.9%
Niger	Africa	-9.5% - -3.2%	-14.4% - -6.8%	-10.9% - 0.0%	-16.1% - 2.2%	11.6% - 65.5%	-20.9% - 54.5%
Nigeria	Africa	-1.5% - 3.4%	-17.3% - -3.3%	-4.5% - 13.7%	-10.5% - 14.3%	5.7% - 100.7%	-54.4% - 105.3%
Rwanda	Africa	-2.1% - 4.5%	-8.4% - 7.0%	-4.4% - 3.6%	-13.0% - 5.5%	-26.4% - 19.6%	-51.9% - 196.7%
Senegal	Africa	-4.1% - 0.9%	-30.4% - -4.5%	-6.9% - 12.5%	-13.0% - 12.2%	-5.9% - 47.1%	-20.9% - 51.3%
Sierra Leone	Africa	-4.3% - 0.8%	-15.0% - 4.1%	-8.2% - 33.4%	-18.9% - 45.6%	0.3% - 84.1%	-9.3% - 240.0%
Somalia	Africa	-4.8% - 1.1%	-51.7% - -0.5%	-5.2% - 15.1%	-25.7% - 18.9%	-3.1% - 8.1%	-4.4% - 10.5%
South Africa	Africa	6.3% - 18.5%	-20.7% - 21.1%	-3.4% - 8.0%	-31.7% - 5.1%	-2.8% - 25.4%	-7.5% - 80.3%
Sudan	Africa	-10.9% - 2.4%	-69.5% - 0.4%	-4.7% - 3.6%	-37.8% - 3.5%	11.9% - 23.9%	-7.3% - 42.5%

Swaziland	Africa	-7.0% - 7.8%	-26.8% - 10.4%	-15.1% - -0.2%	-41.6% - -0.7%	-5.4% - 47.0%	-15.4% - 58.6%
Tanzania	Africa	1.2% - 6.9%	-8.2% - 7.2%	-2.2% - 14.1%	-13.4% - 17.6%	-24.1% - 4.7%	-55.8% - -8.9%
Togo	Africa	-2.8% - 2.2%	-13.5% - -3.7%	-5.5% - 17.3%	-11.2% - 21.0%	20.9% - 226.6%	-17.0% - 692.8%
Tunisia	Africa	-21.5% - 10.2%	-7.7% - 23.4%	-6.3% - 0.3%	-15.2% - -1.4%	-15.6% - 81.0%	-32.5% - 60.0%
Uganda	Africa	1.1% - 6.3%	-17.4% - 0.6%	-4.0% - 15.1%	-16.5% - 17.4%	-17.9% - 53.1%	-71.2% - 199.4%
Zaire	Africa	-6.9% - -0.7%	-21.1% - -2.3%	-5.4% - 7.6%	-9.5% - 6.8%	-25.2% - -3.7%	-65.5% - 2.5%
Zambia	Africa	2.1% - 13.2%	-3.1% - 15.4%	3.2% - 14.0%	-5.3% - 27.1%	-35.0% - -7.3%	-76.0% - -6.2%
Zimbabwe	Africa	-9.7% - 19.7%	-22.6% - 26.6%	-7.3% - 7.8%	-25.9% - 3.0%	-12.5% - 11.4%	-45.7% - 25.8%
Afghanistan	Asia	3.3% - 14.7%	9.6% - 28.8%	-2.3% - -0.3%	-14.2% - 2.2%	-4.9% - 8.8%	-32.9% - 4.5%
Armenia	Asia	3.0% - 11.6%	-1.0% - 10.7%	-5.0% - 6.6%	-16.1% - 3.9%	-6.8% - 14.9%	1.2% - 36.7%
Azerbaijan	Asia	0.0% - 4.5%	4.4% - 12.3%	-2.7% - 5.2%	-14.7% - 2.6%	-12.0% - 5.5%	-16.1% - 14.0%
Bangladesh	Asia	-9.6% - 7.2%	-11.3% - 13.2%	-5.6% - 29.7%	-21.5% - 37.8%	-10.0% - 50.4%	-28.5% - 82.7%
Bhutan	Asia	8.4% - 11.3%	13.2% - 36.5%	-1.1% - 22.4%	3.8% - 39.2%	-29.7% - 9.1%	-35.1% - 48.0%
Brunei	Asia	1.9% - 9.0%	-9.4% - 13.4%	-3.9% - 23.0%	-16.3% - 34.6%	-99.7% - 275.5%	-98.9% - 1269.6%
Cambodia	Asia	1.3% - 8.2%	-15.8% - 7.8%	-3.5% - 27.8%	-11.5% - 33.8%	47.5% - 424.7%	145.7% - 700.4%
China	Asia	-1.1% - 6.6%	-19.8% - 14.0%	-2.9% - 13.9%	-15.8% - 20.7%	-14.4% - 20.1%	-34.1% - 27.7%
Georgia	Asia	-2.9% - 3.2%	-19.2% - -0.2%	-1.8% - 7.5%	-20.7% - 2.6%	2.3% - 24.5%	-14.3% - 113.8%
India	Asia	-12.9% - 1.2%	-33.4% - -7.5%	-13.0% - 18.4%	-28.2% - 22.3%	-16.4% - 47.7%	-38.0% - 56.4%
Indonesia	Asia	0.3% - 4.4%	-7.0% - 6.4%	-7.5% - 16.6%	-15.5% - 23.3%	11.8% - 61.5%	4.1% - 114.2%
Iran	Asia	-3.3% - 9.6%	5.5% - 26.1%	-1.9% - 1.2%	-12.1% - -0.5%	-4.6% - 7.8%	-24.6% - 5.2%
Iraq	Asia	-15.0% - 5.7%	-27.7% - 6.1%	-5.1% - -2.1%	-21.0% - -3.3%	-1.9% - 19.7%	-18.1% - 9.6%
Israel	Asia	-33.5% - 12.3%	-38.9% - 11.1%	-11.1% - 3.9%	-31.5% - -1.3%	-26.1% - 108.3%	-21.0% - 113.4%
Japan	Asia	0.3% - 11.1%	0.1% - 39.3%	-2.9% - 17.3%	-4.3% - 31.4%	-46.5% - 14.3%	-48.7% - 105.4%
Jordan	Asia	-8.6% - 18.9%	-9.6% - 20.8%	-3.2% - 7.0%	-17.7% - -0.3%	-21.8% - 28.4%	-25.0% - 36.9%
Kazakhstan	Asia	-15.2% - 13.8%	-36.5% - 89.5%	-8.3% - 6.2%	-28.9% - 30.8%	-9.0% - 23.0%	-72.7% - 2.5%
Kuwait	Asia	-57.2% - 3.4%	-60.1% - 5.1%	-11.6% - -0.1%	-28.2% - -6.6%	0.0% - 0.0%	0.0% - 0.0%
Kyrgyzstan	Asia	5.4% - 18.3%	4.8% - 39.4%	1.9% - 8.4%	-9.4% - 13.6%	-15.0% - 12.2%	-41.7% - 9.1%
Laos	Asia	-0.4% - 6.1%	-10.9% - 12.7%	-3.5% - 33.0%	-10.8% - 40.9%	-67.4% - 48.8%	-72.0% - 88.0%
Lebanon	Asia	-18.9% - 33.6%	-14.1% - 56.0%	3.0% - 15.6%	-1.0% - 8.9%	-40.8% - 25.5%	-43.3% - 46.2%
Malaysia	Asia	1.0% - 9.3%	-9.2% - 13.4%	-3.4% - 24.0%	-15.1% - 32.5%	-33.0% - 81.7%	-53.9% - 239.6%

Mongolia	Asia	1.5% - 8.8%	-11.0% - 17.7%	-4.8% - 3.4%	-19.9% - 10.2%	-3.3% - 33.6%	-70.0% - 36.3%
Myanmar	Asia	0.1% - 8.7%	-10.5% - 12.0%	-2.8% - 43.5%	-11.1% - 45.1%	-19.2% - 29.4%	-52.6% - 72.8%
Nepal	Asia	-48.9% - 0.3%	-56.3% - -1.7%	-50.0% - 20.4%	-58.1% - 23.1%	-37.8% - 13.5%	-61.1% - 45.5%
North Korea	Asia	-2.6% - 8.5%	-15.1% - 20.5%	-1.2% - 17.2%	-6.1% - 28.9%	-25.9% - 18.0%	-40.4% - 17.1%
Oman	Asia	-71.7% - 1.2%	-76.5% - -18.3%	-12.4% - -3.0%	-32.7% - -13.2%	-4.1% - 12.0%	-15.5% - 14.2%
Pakistan	Asia	-21.4% - 3.7%	-57.2% - -6.8%	-15.6% - 6.4%	-36.3% - 8.4%	-11.4% - 10.8%	-34.5% - 23.0%
Philippines	Asia	-1.5% - 1.5%	-8.9% - 2.3%	-3.9% - 23.5%	-10.8% - 28.4%	-29.6% - 117.7%	-37.2% - 199.1%
Qatar	Asia	-11.2% - 0.3%	-91.8% - -5.2%	4.6% - 27.9%	-88.0% - 31.7%	0.0% - 0.0%	0.0% - 0.0%
Saudi Arabia	Asia	-55.6% - 7.7%	-59.5% - -5.5%	-8.9% - 5.7%	-20.1% - 0.3%	-1.9% - 4.4%	-3.6% - 6.4%
South Korea	Asia	-1.4% - 23.3%	36.2% - 63.7%	-1.8% - 15.0%	-19.3% - 19.9%	-28.1% - -6.1%	-50.3% - -7.0%
Sri Lanka	Asia	-7.0% - 0.6%	-15.0% - 3.1%	-3.6% - 27.7%	-10.3% - 41.7%	-15.6% - 67.5%	-68.8% - 47.1%
Syria	Asia	-3.3% - 15.2%	-16.2% - 16.1%	-1.6% - 6.0%	-24.1% - 3.0%	-20.0% - 10.2%	-18.7% - 23.2%
Tajikistan	Asia	1.8% - 21.0%	11.6% - 55.6%	-3.7% - 2.3%	-5.1% - 1.7%	-16.7% - 16.3%	-56.9% - 12.7%
Thailand	Asia	-1.4% - 7.5%	-16.8% - 3.7%	-3.8% - 22.9%	-11.3% - 27.1%	-33.1% - 46.0%	-32.0% - 170.4%
Turkey	Asia	-4.1% - 8.8%	-1.5% - 16.1%	-1.6% - 2.9%	-15.0% - 1.8%	-4.0% - 22.8%	-4.4% - 27.0%
Turkmenistan	Asia	0.6% - 18.5%	-7.1% - 26.2%	-2.5% - 0.0%	-21.2% - -2.2%	-14.1% - -1.3%	-25.1% - 3.8%
United Arab Emirates	Asia	-47.1% - -2.1%	-57.0% - -20.1%	-7.0% - 13.1%	-35.4% - 5.1%	-1.0% - 26.4%	-18.0% - 34.1%
Uzbekistan	Asia	-3.6% - 11.7%	0.5% - 20.0%	-5.4% - -2.6%	-19.9% - -0.6%	-12.9% - 10.0%	-30.4% - -2.7%
Vietnam	Asia	-1.9% - 7.0%	-21.1% - 5.5%	-3.4% - 29.8%	-12.4% - 39.8%	-31.6% - 100.7%	-6.5% - 273.9%
West Bank	Asia	-39.2% - 30.4%	-39.6% - 37.4%	-13.2% - 10.8%	-15.5% - 11.6%	-39.4% - 88.1%	-54.1% - 96.4%
Yemen	Asia	-6.4% - 1.7%	-41.9% - 9.2%	-12.5% - 8.0%	-54.6% - 19.9%	0.3% - 19.4%	-42.9% - 11.0%
Albania	Europe	-6.7% - 1.8%	-28.1% - 5.7%	-3.9% - 3.7%	-32.7% - 7.6%	11.3% - 72.0%	-12.8% - 45.7%
Austria	Europe	-4.1% - 8.7%	-4.4% - 25.2%	-2.8% - 5.3%	-16.2% - 9.8%	6.7% - 68.1%	-37.8% - 148.3%
Belgium	Europe	-2.3% - 7.7%	-18.1% - 29.2%	-6.3% - 10.6%	-30.3% - 15.3%	-26.4% - 64.2%	-23.7% - 274.5%
Bosnia & Herzegovina	Europe	-8.1% - 3.7%	-26.1% - 11.8%	-4.6% - 4.2%	-25.7% - 3.1%	8.2% - 34.0%	0.0% - 85.2%
Bulgaria	Europe	-13.8% - -1.1%	-13.5% - 0.3%	-9.0% - -0.7%	-21.0% - -2.8%	13.4% - 51.6%	-16.4% - 44.5%
Byelarus	Europe	10.8% - 23.8%	2.4% - 39.4%	6.7% - 21.8%	-10.0% - 27.3%	-35.1% - 31.7%	-79.6% - 161.1%
Croatia	Europe	-12.3% - 1.6%	-32.1% - 3.4%	-11.6% - 0.9%	-29.1% - -1.1%	6.0% - 52.1%	-61.3% - 114.4%
Cyprus	Europe	-13.7% - -4.5%	-13.3% - 4.3%	-3.8% - 18.8%	-17.4% - 20.8%	-13.9% - 1.7%	-21.2% - -0.2%
Czech Republic	Europe	0.0% - 11.0%	3.3% - 23.9%	1.9% - 10.0%	-6.7% - 11.6%	-37.7% - 19.0%	-48.5% - 100.0%

Denmark	Europe	9.0% - 20.8%	-0.3% - 25.8%	3.0% - 16.6%	-6.7% - 13.8%	-50.7% - -9.3%	-34.1% - 51.0%
Estonia	Europe	4.3% - 11.4%	0.1% - 15.3%	4.9% - 14.9%	-13.2% - 13.1%	-14.1% - 75.7%	-31.6% - 143.4%
Finland	Europe	4.7% - 19.3%	-16.1% - 27.2%	-1.8% - 10.2%	-25.3% - 4.6%	-44.1% - -4.5%	-43.9% - 99.4%
France	Europe	-8.1% - 0.7%	-23.4% - 11.0%	-5.3% - 10.8%	-23.9% - 13.0%	-11.2% - 47.8%	3.7% - 140.7%
Germany	Europe	1.4% - 12.8%	2.1% - 36.1%	-1.1% - 12.0%	-16.1% - 15.8%	-14.3% - 78.0%	-82.5% - 379.3%
Greece	Europe	-15.1% - -6.1%	-17.4% - 13.5%	-11.2% - -2.1%	-19.3% - 4.3%	19.7% - 57.2%	-11.4% - 45.4%
Hungary	Europe	-7.3% - 6.4%	-24.0% - 4.5%	-7.9% - 5.7%	-30.4% - 3.7%	-7.4% - 25.2%	-13.7% - 56.7%
Ireland	Europe	-1.8% - 5.7%	3.5% - 11.6%	-6.7% - 9.5%	-20.2% - 17.4%	0.0% - 0.0%	0.0% - 0.0%
Italy	Europe	-6.6% - 2.4%	-25.4% - 6.7%	-3.1% - 8.7%	-13.2% - 12.0%	-9.1% - 42.4%	-19.5% - 68.0%
Latvia	Europe	-2.1% - 8.3%	-2.0% - 10.1%	1.3% - 11.5%	-8.8% - 5.7%	-18.8% - 70.4%	-32.5% - 173.4%
Lithuania	Europe	4.8% - 12.3%	-2.7% - 15.3%	4.7% - 14.1%	-8.4% - 8.4%	-56.5% - -16.1%	-49.5% - 84.7%
Luxembourg	Europe	-3.0% - 16.0%	-1.3% - 25.6%	-6.8% - 13.4%	-40.7% - 11.8%	-33.8% - 65.5%	0.0% - 265.0%
Macedonia	Europe	-17.3% - -8.0%	-15.2% - 14.6%	-9.4% - -2.6%	-22.4% - 4.4%	16.0% - 37.8%	-4.4% - 55.8%
Moldova	Europe	-5.8% - 6.8%	-21.6% - 3.5%	-3.3% - 12.9%	-23.9% - 11.2%	-15.5% - 14.1%	-34.1% - 41.9%
Montenegro	Europe	-8.8% - 11.6%	-8.0% - 33.1%	1.4% - 5.8%	-14.3% - 12.8%	35.8% - 134.6%	-88.6% - 222.8%
Netherlands	Europe	-0.2% - 10.3%	4.8% - 44.9%	-4.1% - 10.5%	-16.1% - 22.3%	-12.8% - 68.8%	0.0% - 340.9%
Norway	Europe	3.3% - 18.1%	-3.8% - 30.4%	0.7% - 9.3%	-10.3% - 10.7%	0.0% - 0.0%	0.0% - 0.0%
Poland	Europe	3.3% - 11.7%	4.2% - 26.0%	0.2% - 12.1%	-8.4% - 16.1%	-36.1% - 2.0%	-36.3% - 36.6%
Portugal	Europe	-8.9% - 1.6%	-47.0% - 0.5%	-6.5% - 3.2%	-28.9% - 7.0%	-2.1% - 75.5%	-2.7% - 76.5%
Romania	Europe	-6.7% - 3.3%	-24.2% - 0.1%	-6.3% - 5.0%	-27.1% - 0.3%	0.8% - 26.7%	-33.4% - 46.1%
Russia	Europe	1.5% - 17.1%	-6.0% - 35.6%	-2.6% - 9.3%	-13.6% - 16.1%	-24.3% - 21.0%	-65.4% - 26.1%
Serbia	Europe	-13.9% - 0.2%	-30.8% - -0.5%	-9.2% - 1.5%	-26.9% - -1.7%	12.3% - 45.7%	-67.3% - 75.7%
Slovakia	Europe	-0.7% - 8.3%	-9.0% - 14.0%	-3.8% - 7.1%	-20.3% - 13.6%	-11.3% - 36.5%	-10.9% - 98.4%
Slovenia	Europe	-1.7% - 6.1%	-10.0% - 11.8%	-3.9% - 3.1%	-17.7% - 7.2%	11.8% - 77.6%	-69.1% - 163.2%
Spain	Europe	-7.4% - 8.5%	-18.7% - 14.3%	-3.2% - 2.3%	-21.9% - -0.2%	-7.1% - 28.5%	-10.3% - 61.3%
Sweden	Europe	2.7% - 16.4%	-8.7% - 23.7%	-5.0% - 6.7%	-25.5% - 4.9%	0.0% - 0.0%	0.0% - 0.0%
Switzerland	Europe	-4.5% - 9.0%	3.9% - 29.7%	-2.7% - 6.1%	-6.5% - 15.1%	15.8% - 136.1%	24.2% - 592.1%
Ukraine	Europe	0.7% - 13.1%	-6.2% - 17.1%	-0.6% - 16.3%	-13.7% - 18.7%	-25.9% - 13.8%	-35.8% - 48.9%
United Kingdom	Europe	0.7% - 11.0%	2.9% - 24.0%	-3.6% - 12.9%	-17.7% - 18.3%	-28.2% - 16.9%	-40.1% - 49.9%
Canada	N. America	-16.4% - -2.0%	0.4% - 25.7%	-8.5% - -2.7%	-8.5% - 12.4%	16.7% - 57.7%	-42.7% - 29.4%

Costa Rica	N. America	0.4% - 7.8%	-18.6% - 10.9%	-6.5% - 19.5%	-18.6% - 22.2%	-23.8% - 177.2%	-28.9% - 510.0%
Cuba	N. America	-4.3% - 17.4%	0.0% - 22.0%	-4.3% - 60.1%	0.0% - 64.5%	0.0% - 0.0%	0.0% - 0.0%
Dominican Republic	N. America	3.5% - 10.6%	-6.9% - 9.0%	-2.8% - 26.2%	-17.2% - 27.7%	-24.1% - 48.2%	-51.0% - 63.2%
El Salvador	N. America	-3.2% - 5.0%	-23.2% - 7.2%	-4.7% - 18.8%	-17.4% - 22.3%	-5.8% - 80.6%	-62.7% - 127.7%
Guadeloupe	N. America	-5.2% - -1.9%	0.0% - 3.5%	-1.9% - 28.7%	0.0% - 37.1%	0.0% - 0.0%	0.0% - 0.0%
Guatemala	N. America	-3.4% - 4.8%	-27.6% - 2.1%	-4.0% - 21.4%	-13.4% - 24.3%	-0.3% - 28.2%	-37.1% - 37.5%
Haiti	N. America	3.6% - 10.0%	-9.8% - 4.9%	-1.5% - 31.3%	-17.8% - 29.1%	-49.2% - 105.2%	-62.9% - 165.2%
Honduras	N. America	-4.3% - 4.7%	-28.4% - 3.1%	-1.1% - 23.1%	-15.9% - 24.1%	-29.1% - 83.7%	-33.7% - 186.3%
Mexico	N. America	-3.8% - 4.3%	-34.0% - 0.1%	-6.6% - 9.7%	-23.6% - 8.8%	-10.3% - 44.8%	-35.1% - 53.8%
Nicaragua	N. America	-2.0% - 6.8%	-29.2% - 7.2%	-3.7% - 23.9%	-16.8% - 26.8%	4.0% - 118.0%	-33.4% - 147.5%
Puerto Rico	N. America	-8.7% - -4.0%	0.0% - 2.4%	-3.7% - 24.4%	0.0% - 34.1%	0.0% - 0.0%	0.0% - 0.0%
St. Vincent & Grenadines	N. America	-4.2% - 3.0%	-9.1% - 9.3%	-1.8% - 20.2%	-9.0% - 23.8%	0.0% - 0.0%	0.0% - 0.0%
Trinidad and Tobago	N. America	-6.8% - -4.0%	-5.2% - 5.7%	-6.1% - 29.5%	-14.1% - 29.0%	-55.8% - 144.2%	-18.7% - 246.6%
United States	N. America	-11.2% - 1.5%	-21.9% - 7.5%	-7.6% - 1.4%	-21.6% - 5.2%	12.9% - 40.4%	-48.8% - 35.6%
Australia	Oceania	-15.7% - 1.4%	-19.3% - 13.2%	-15.2% - -4.6%	-27.1% - 2.0%	12.5% - 39.5%	-6.3% - 62.4%
Fiji	Oceania	-7.2% - 1.0%	-13.1% - 10.0%	-11.8% - 10.2%	-10.9% - 16.7%	0.0% - 0.0%	0.0% - 0.0%
New Caledonia	Oceania	-5.5% - 20.5%	-10.8% - 8.1%	-12.9% - 85.6%	-25.2% - 78.3%	0.0% - 0.0%	0.0% - 0.0%
New Zealand	Oceania	13.0% - 17.8%	14.9% - 36.9%	9.1% - 23.9%	-4.8% - 23.6%	-7.1% - 8.4%	0.0% - 78.5%
Papua New Guinea	Oceania	-8.1% - -2.2%	-22.8% - 0.6%	-1.3% - 17.9%	-11.6% - 18.1%	0.0% - 0.0%	0.0% - 0.0%
Vanuatu	Oceania	-10.6% - -4.3%	-22.0% - -5.6%	-2.2% - 27.4%	-14.7% - 42.1%	0.0% - 0.0%	0.0% - 0.0%
Argentina	S. America	-2.8% - 11.0%	-7.2% - 13.7%	-1.6% - 1.9%	-4.4% - 0.9%	10.0% - 65.8%	-54.4% - 117.1%
Belize	S. America	0.9% - 5.8%	-20.8% - -1.5%	-3.4% - 23.3%	-15.9% - 20.6%	-41.3% - 42.4%	-92.5% - 181.9%
Bolivia	S. America	-5.0% - 4.9%	-16.2% - 11.0%	0.4% - 5.2%	-11.4% - 7.1%	-16.5% - 3.6%	-59.4% - 54.1%
Brazil	S. America	-1.6% - 5.5%	-22.4% - 6.2%	-4.8% - 7.5%	-23.0% - 5.9%	-2.1% - 70.1%	-67.4% - 89.9%
Chile	S. America	6.0% - 16.9%	12.6% - 48.9%	4.8% - 12.1%	5.1% - 32.1%	8.9% - 43.7%	-39.7% - 59.3%
Colombia	S. America	-2.7% - 2.9%	-23.9% - 0.3%	-2.2% - 19.8%	-21.0% - 20.4%	-6.7% - 43.2%	-6.6% - 196.5%
Ecuador	S. America	2.7% - 8.7%	-24.5% - 12.8%	0.0% - 11.6%	-11.3% - 6.9%	-5.4% - 10.9%	-16.2% - 25.4%
French Guiana	S. America	2.2% - 5.9%	-2.5% - 3.7%	-7.6% - 28.1%	-27.3% - 36.0%	141.3% - 2853.0%	116.9% - 8415.5%
Guyana	S. America	0.7% - 7.5%	-5.0% - 12.1%	-4.0% - 39.6%	-18.7% - 46.5%	41.7% - 194.1%	28.9% - 813.5%
Jamaica	S. America	-8.1% - -0.4%	-12.4% - 0.5%	-3.3% - 19.0%	-13.5% - 18.4%	0.0% - 0.0%	0.0% - 0.0%

Panama	S. America	-1.3% - 3.3%	-20.0% - 2.5%	-2.6% - 27.4%	-12.1% - 36.4%	-4.4% - 346.0%	-31.3% - 855.3%
Paraguay	S. America	-8.2% - -2.8%	-33.0% - -7.5%	-11.4% - 1.7%	-44.1% - -3.9%	-10.2% - 90.1%	-12.8% - 358.2%
Peru	S. America	4.1% - 19.0%	1.8% - 44.9%	1.5% - 10.0%	0.4% - 26.9%	-8.2% - 1.9%	-28.5% - 1.9%
Suriname	S. America	1.2% - 9.0%	-4.7% - 18.3%	-6.5% - 40.0%	-21.6% - 44.5%	76.1% - 1289.5%	142.4% - 4155.3%
Uruguay	S. America	0.8% - 11.8%	3.9% - 29.5%	5.8% - 13.6%	12.9% - 20.1%	-19.5% - 51.4%	12.4% - 161.4%
Venezuela	S. America	-7.7% - 2.6%	-42.2% - -2.8%	-4.2% - 23.0%	-22.6% - 25.1%	-15.0% - 35.2%	-13.9% - 178.4%
